# Novel Biochemical Markers of Psychosocial Stress in Women

**DOI:** 10.1371/journal.pone.0003590

**Published:** 2009-01-29

**Authors:** Marie Åsberg, Åke Nygren, Rosario Leopardi, Gunnar Rylander, Ulla Peterson, Lukas Wilczek, Håkan Källmén, Mirjam Ekstedt, Torbjörn Åkerstedt, Mats Lekander, Rolf Ekman

**Affiliations:** 1 Department of Clinical Sciences, Karolinska Institute, Stockholm, Sweden; 2 Department of Clinical Neuroscience, Karolinska Institute, Stockholm, Sweden; 3 Institute for Psychosocial Medicine and Department of Public Health, Karolinska Institute, Stockholm, Sweden; 4 Institute of Clinical Neuroscience and Physiology, Göteborg University, Göteborg, Sweden; James Cook University, Australia

## Abstract

**Background:**

Prolonged psychosocial stress is a condition assessed through self-reports. Here we aimed to identify biochemical markers for screening and early intervention in women.

**Methods:**

Plasma concentrations of interleukin (IL) 1-α, IL1-β, IL-2, IL-4, IL-6, IL-8, IL-10, interferon-γ (INF-γ), tumor necrosis factor-α (TNF-α), monocyte chemotactic protein-1 (MCP-1), epidermal growth factor (EGF), vascular endothelial growth factor (VEGF), thyroid stimulating hormone (TSH), total tri-iodothyronine (TT3), total thyroxine (TT4), prolactin, and testosterone were measured in: 195 women on long-term sick-leave for a stress-related affective disorder, 45 women at risk for professional burnout, and 84 healthy women.

**Results:**

We found significantly increased levels of MCP-1, VEGF and EGF in women exposed to prolonged psychosocial stress. Statistical analysis indicates that they independently associate with a significant risk for being classified as ill.

**Conclusions:**

MCP-1, EGF, and VEGF are potential markers for screening and early intervention in women under prolonged psychosocial stress.

## Introduction

Sustained psychosocial stress, often related to work, is an increasingly important factor in the development of illness, physical as well as mental [1]–[3]. In Sweden, public expenditure for sick-leave more than doubled in a few years, and in 2003 the number of workers on long-term sick-leave (more than 30 days) increased to all time high levels [4]. Women represent about 70–80% of this patient group. We have studied more than 400 patients on long-term sick leave because of an affective disorder, and found that about 80% met DSM-IV criteria for major depression at some time during their illness (our unpublished data). However, mental and physical exhaustion were the most prominent symptoms, which tended to persist after the depressive symptoms had cleared. Many patients remained incapacitated for a very long period with a pronounced tendency to recurrence.

The sequence of pathophysiological events set forward by prolonged exposure to mental stress in humans is not completely characterized, but it involves the hypothalamus-pituitary-adrenal (HPA) axis, as well as the endocrine and the immune systems [5]–[7]. The cortisol response to corticotropin-releasing hormone (CRH) was recently analyzed in our patients [8]. The results showed an attenuated dexamethasone-CRH response, a feature opposite to that seen in other patients with major depression [9]. Such a finding suggests an imbalance in other physiological systems. Metabolic processes occurring under chronic stress might be reflected in altered hormones, cytokines and cellular growth factors levels [10]–[11].

Here, we have taken advantage of the microarray technology, which uses miniaturized microspot ligand-binding assays to enable the simultaneous measurement of a large number of biochemical markers from a minute plasma sample within a single assay [12]. Using this technique we assessed a panel of 17 biological mediators (hormones, cytokines, and cellular growth factors), aiming to investigate whether plasma concentrations of any of these molecules could be related to prolonged psychosocial stress, and if so, whether they could be used, individually or in combination, as markers for screening and diagnostic purposes.

## Materials and Methods

### Patients on long term sick leave

The studies were cleared by the Research Ethics Committees of the Karolinska Institute, and the Medical Faculty of Linköping University, respectively. One hundred ninety-five women (mean age 44, SD = 9.1 years, age range 21–60 years) on long-term sickleave (more than three months) for any affective or stress-related mental disorder (depression, anxiety disorder, stress disorder, burnout syndrome, exhaustion disorder) were recruited from one of the major Swedish insurance companies, the Alecta Company, which insures about 600,000 employees in private enterprises, mainly white collar workers. The company keeps a database which includes diagnostic information for all cases of sick leave exceeding 3 months. Letters were sent to all patients on sick leave with any of the above mentioned diagnoses on their sickness certificate. Patients were then approached by telephone and invited to participate in a clinical study involving interviews and questionnaires, as well as an offer of further treatment. Written informed consent was obtained from all participants. All patients were ambulatory and none had received inpatient care for their current illness. They were diagnosed according to the Diagnostic and Statistical Manual of the American Psychiatric Association, 4th Edition [DSM–IV; 13] by specially trained physicians, using the Structured Clinical Interview for DSM-IV [14]. Eighty-two per cent met DSM-IV criteria for Major Depressive Disorder at some time during their current illness episode. Likely eliciting factors were: work-related stress (39%), stressful family relationships (9.3%), a combination of work and family stressors (49.3%), or not identified (2.1%).

### Health care personnel with occupational stress

A group of women experiencing work stress was selected from the results of a questionnaire sent to all 6118 health care employees of a Swedish county council (Kalmar). Of the 3976 employees who replied to the questionnaire, those who scored above the 75th percentile on the Oldenburg Burnout Inventory [OLBI; 15], which measures degree of professional burnout, were invited to participate in a randomized controlled study of the possible beneficial effect of a series of structured group discussions with colleagues. Those who were randomised to active treatment were asked to leave blood samples prior to treatment. The resulting group consisted of 45 women, ranging in age from 39 to 62 years, mean age 52.8, SD = 5.2 years.

### Healthy control workers

The control group included 84 women (mean age 36.1, SD = 8.4, range 23–62 years), recruited among the employees of a Swedish IT-company. Out of 560 employees, both women and men, the above 84 individuals had agreed to undergo a physiological examination as part of a health screening in a stress prevention program at the company. The subjects were all full time workers, of whom 34% were managers and 54% project leaders. Two women who were pregnant at the time of the physiological examination were excluded.

### Analytical methods

Venous blood was drawn into tubes containing EDTA, and immediately centrifuged. Plasma was separated and stored in aliquots at −20°C or below until analyzed. The following cytokines and growth factors were analyzed: interleukin 1-α (IL1-α), interleukin 1-β (IL1-β), interleukin 2 (IL-2), interleukin 4 (IL-4), interleukin 6 (IL-6), interleukin 8 (IL-8), interleukin 10 (IL-10), interferon-γ (INF-γ), tumor necrosis factor-α (TNF-α), monocyte chemotactic protein-1 (MCP-1), epidermal growth factor (EGF), and vascular endothelial growth factor (VEGF). Furthermore, thyroid stimulating hormone (TSH), total tri-iodothyronine (TT3), total thyroxine (TT4), prolactin, and testosterone were included in the panel analyzed on a high throughput automated biochip immunoassay system, Evidence®, Randox Laboratories Ltd [Crumlin, UK; 16]

### Statistical analysis

All analyses were performed with the SPSS 11.5 for Windows. One-way ANOVA's were calculated by using the group variable as independent variable with 3 levels, and each biochemical marker as a dependent variable. The significance levels were adjusted according to Bonferroni's method. Pair-wise *post hoc* comparisons were made according to Scheffe's method. To determine the optimal cut-off point maximizing the sensitivity (true positive rate) and specificity (true negative rate), receiver operating curve (ROC) analyses were performed [17]. An area under the ROC curve of 0.9 or above is needed for a reliable differentiation of the groups. To calculate the odds of being classified as ill or not, when having a value above, compared with below the established cut-off, logistic regression analyses were used.

## Results

Means and standard deviations for the 17 markers in the three groups are shown in Tables 1 and 2. Neither the interleukins nor IFN-γ or TNF-α differed between the groups. Differences were observed, however, between the three subject groups for MCP-1, EGF, and VEGF. MCP-1 levels were more than twice as high in the sick leave group compared to the healthy controls, with the occupational stress group in between. VEGF levels were three times as high in the sick leave group, and EGF levels were more than twice as high, compared to the healthy group, once again with the occupational stress group in between. The sick leave group also had significantly lower levels of prolactin and TSH (Table 1).

**Table 1:** Biochemical markers in women experiencing different levels of stress.

Marker	Sick leave (group 1)		Occupational stress (group 2)		Healthy Subjects (group 3)		ANOVA			Significant group pair wise comparisons
	M	SD	M	SD	M	SD	F	Df	P	
IL1-α	5.1	14.1	5.6	6.1	5.4	5.1	0.92	2,338	.402	
IL1-β	2.9	6.4	2.5	4.0	2.1	2.7	0.59	2,338	.554	
IL-2	17.6	39.4	18.6	24.0	14.6	24.0	0.31	2,338	.734	
IL-4	3.8	3.5	3.2	0.8	3.3	1.7	1.82	2,338	.163	
IL-6	6.6	16.4	9.4	26.2	13.7	33.4	2.87	2,333	.058	
IL-8	5.4	10.8	3.3	2.9	3.1	1.3	2.98	2,338	.052	
IL-10	3.4	6.6	3.0	5.6	6.5	20.3	2.37	2,338	.095	
IFN-γ	0.7	0.7	0.7	0.7	0.7	0.7	0.39	2,338	.678	
TNF-α	5.1	16.2	3.3	2.5	5.3	19.6	0.26	2,338	.773	
MCP-1	348.4	126.7	217.8	92.6	160.2	85.7	97.82	2,338	.000*	1–2, 1–3, 2–3
EGF	117.0	77.2	70.6	53.0	29.4	47.5	56.16	2,338	.000*	1–2, 1–3, 2–3
VEGF	30.9	22.7	18.4	15.4	10.3	7.1	41.24	2,338	.000*	1–2, 1–3
Prolactin	388.6	216.4	534.0	246.3	684.1	748.0	15.47	2,346	.000*	1–3
TT3	0.5	0.3	0.6	0.2	0.5	0.3	6.46	2,329	.002*	1–2
TT4	6.9	1.6	7.1	1.6	7.2	1.8	0.82	2,441	.442	
TSH	1.8	1.0	2.4	1.6	2.4	1.2	11.11	2,342	.000*	1–3
Testosterone	4.1	1.5	4.0	1.8	3.5	1.4	5.39	2,330	.005	

Concentration is given in pg/ml except for prolactin (mIU/L), TT3 (ng/ml), TT4 (g/L), and TSH (μIU/ml), and testosterone (nmol/L). Mean (M) and standard deviation (SD) values are indicated for the three test groups. Significant comparisons between groups (P<.05 level) are indicated in the last column.

**Table 2:** Optimal cut-off, area under the ROC-curve (Area), and diagnostic sensitivity and specificity of statistically significant biochemical markers.

Marker	Area	Cut-off	Sensitivity	Specificity
MCP-1	0.886	243.00	0.85	0.92
VEGF	0.805	7.80	0.78	0.85
EGF	0.798	68.00	0.69	1.00
Prolactin	0.699	380.00	0.60	0.79
TSH	0.624	1.80	0.58	0.69
Testosterone	0.618	3.73	0.63	0.65

Since the correlations between some of the markers and age were significant, and the mean age differed significantly between the groups (one-way ANOVA F = 59.09 df = 2,338, P = 0.000), we controlled for age in an analysis of covariance. This resulted in one additional significant difference, namely in testosterone, which was higher in the sick leave group. In order to examine the usefulness of these markers for screening and diagnostic purposes, a receiver operating characteristic (ROC) curve analysis was performed (Table 2). As seen from the table, the best sensitivity and specificity was obtained for MCP-1, VEGF, and EGF.

The relative value of MCP-1, EGF, and VEGF as risk factors for classification as ill or healthy was also tested. The results are shown in Table 3, and indicate that each of these markers independently associates with a significantly increased risk for being classified as ill.

**Table 3:** Relative risks of being classified as ill, using the established cut-off points of MCP-1, EGF and VEGF (Table 3).

Marker	Beta	Wald	P	OR	95% CI
MCP-1	3.55	52.17	0.000	34.85	13.30–91.35
EGF	2.12	18.02	0.000	8.35	3.13–22.25
VEGF	2.08	18.99	0.000	8.02	3.14–20.44

Results from multiple logistic regression analyses are shown. OR, Observed Risk, (95% CI).

## Discussion

Our results show a direct correlation between plasma concentrations of MCP-1, EGF, and VEGF, and psychosocial stress-related illness. Plasma levels were elevated in subjects with occupational stress, and more so in a group of subjects on long term sick leave for an affective disorder following exposure to chronic stress. Statistically, each of these three markers associated independently with a significantly increased risk for being classified as ill.

However, our results should be seen as preliminary, and need to be replicated because of a number of intrinsic limitations:

- The study design has involved only one single time point observation during the course of a prolonged stress condition. This design may in principle suffer from possible short time-course variations. However, we have data from an almost complete 2-year follow up study on our recovering patient cohort, showing that MCP-1, EGF, and VEGF in plasma decrease very slowly within months to years (Åsberg et al, unpublished).
- The daytime point for blood withdrawal varied among individuals approximately between 9 AM and 3 PM, making circadian rhythm a possible confounding factor. Circadian rhythms have been described for EGF and VEGF, and MCP-1 [18], [19], [20]. However, both EGF and VEGF plasma levels are relatively stable during daytime [21], [22]. Thus, it is unlikely that collection time points could significantly influence results on these two mediators. On the other hand, the studies available on MCP-1 were done in mice peritoneal macrophages [20], and to our knowledge similar studies have not been confirmed in any human tissue. Thus, it remains entirely possible that collection time points may have played a role for MCP-1, as well as for other markers for which we have not found a significant association with stress and disease. Indeed, several of these markers are known to have circadian rhythms [23], and for this reason our negative results should be taken with caution.
- We have not controlled for nicotine consumption or for sex hormone variables such as menstrual phase or oral contraceptive use. Also, data on nutrition and physical activity have not been consistently collected across groups.

While our study is mainly concerned with their potential use as markers of disease, plasma MCP-1, EGF, and VEGF may also be related to pathophysiological outcomes that are worth to consider. For instance, MCP-1 mediates inflammatory-like disorders and oxidative stress [24], and it also contributes to macrophage infiltration into adipose tissue and insulin resistance [25]. Moreover, at least for acute stress there are examples of an MCP-1 effect on chemotaxis and immune cell redistribution. Similarly, there is a documented relationship between MCP-1 and atherosclerosis. [26]. On the other hand, EGF is a mediator of stress-related events influencing the cell cycle [27]. This might be particularly important in heart diseases, where adverse effects of angiotensin II could be partly mediated by EGF [28]. Similarly, VEGF has been reported to be upregulated by angiotensin II, and thereby activate vascular inflammation [29]. However, VEGF has also been reported to act as a neuroprotective factor, which makes our results somewhat intriguing [30]. Also, VEGF has recently been shown to selectively recruit stem and progenitor cells to specific organs [31].

While psychosocial stress in humans is a complex phenomenon not readily captured by bodily biochemical modifications, animal models have began to shed light on translational mediating events. In mice, psychosocial stress has been shown to be converted into cell cycle signalling, and the mechanism is mediated by the transcription factor nuclear factor kappaB [NF–kappaB; 32]. Interestingly, NF-kappaB expression in humans has been reported to be influenced by EGF and VEGF during various pathological conditions [33], [34]. In turn, MCP-1 expression has been shown to be controlled by NF-kappaB [35]. Taken together, these data suggest that psychosocial stress may broadly influence pathophysiological changes acting on a cellular level also in humans.

Previous studies have shown that patients on sick leave because of occupational burnout resulting from psychosocial stress, have a disrupted sleep, with more arousals and sleep fragmentation, more wake time, and lower sleep efficiency [36]. Sleep fragmentation is associated with elevated levels of metabolic and cardiovascular risk indicators of stress-related disorders, such as morning cortisol, heart rate, systolic and diastolic blood pressure, total cholesterol, high-density lipoprotein (HDL)- and low-density lipoprotein (LDL)-cholesterol, and LDL/HDL-ratio [37]. It is conceivable that some of the pathophysiological changes developing during exposure to psychosocial chronic stress reflect sleep disturbances. Some studies suggest that MCP1, EGF en VEGF levels may indeed be related with sleep quality, and be altered as an effect of disrupted sleep [38]–[40]. It would be therefore important to test a correlation of MCP1, EGF and VEGF levels in relationship to qualitative and quantitative sleep alterations.

Taken together with the recently reported dexamethasone-CRH data [8], our results indicate that women under prolonged psychosocial stress develop so-far unique neuro-endocrine-immune alterations. If confirmed, our results may be developed into novel work hypotheses to construct models for further investigations both in the preclinical and in the clinical settings. Also, our data may bring to the clinician a potential tool for diagnosis of a condition that is poorly understood, not diagnosable through laboratory tests, yet progressively more common in industrialized areas of the word.
